# Recombinant human erythropoietin and interferon-β-1b protect against 3-nitropropionic acid-induced neurotoxicity in rats: possible role of JAK/STAT signaling pathway

**DOI:** 10.1007/s10787-022-00935-x

**Published:** 2022-03-06

**Authors:** Rabab H. Sayed, Amira H. Ghazy, Mohamed F. El Yammany

**Affiliations:** Department of Pharmacology and Toxicology, Faculty of Pharmacy, Cairo University, Kasr El Aini St, Cairo, 11562 Egypt

**Keywords:** Huntington’s disease, 3-Nitropropionic acid, Interferon-beta-1b, Recombinant human erythropoietin, Rats

## Abstract

3-Nitropropionic acid (3-NP) model serves as a beneficial tool to evaluate the effect of novel treatments for Huntington’s disease (HD). The aim of the present study was to demonstrate the neuroprotective effect of recombinant human erythropoietin (rhEPO) and interferon-beta-1b (IFN-β-1b) in 3-NP-induced neurotoxicity in rats. Rats were injected with 3-NP (10 mg/kg/day, i.p) for 2 weeks and were divided into five subgroups; the first served as the HD group, the second received rhEPO (5000 IU/kg/every other day, i.p.) for 2 weeks, the third received rhEPO starting from the 5th day of 3-NP injection, the fourth received IFN-β-1b (300,000 units, every day other day, s.c) for 2 weeks, and the last received IFN-β-1b starting from the 5th day of 3-NP injection. All treatments significantly improved motor and behavior performance of rats. Moreover, all treatments markedly restored mitochondrial function as well as brain-derived neurotrophic factor level, and reduced oxidative stress biomarkers, pro-inflammatory mediators, nuclear factor kappa B expression, caspase-3, and Bax/Bcl2 ratio in the striatum. In conclusion, the present study demonstrates the neuroprotective potential of rhEPO or IFN-β-1b on 3-NP-induced neurotoxicity in rats. Furthermore, our study suggests that activation of JAK2/STAT3 or JAK1/STAT3 may contribute to the neuroprotective activity of rhEPO or IFN-β-1b, respectively. We also found that early treatment with rhEPO did not confer any benefits compared with late rhEPO treatment, while early IFN-β-1b showed a marked significant benefit compared with late IFN-β-1b.

## Introduction

Huntington’s disease (HD) is an incurable autosomal dominant progressive neurodegenerative disease characterized by neuronal degeneration in the striatum and is associated with involuntary movements, cognitive deficits, and psychiatric disturbances (Ayala-Peña 2013; Ramaswamy et al. 2015). 3-Nitropropionic acid (3-NP) is a natural mycotoxin that can readily pass the blood–brain barrier (BBB) and induces dystonic movements and cognitive impairments resembling that of human HD (Kaur et al. 2015). 3-NP causes irreversible inhibition of mitochondrial complex II enzyme, succinate dehydrogenase (SDH), blocking electron transport chain and Krebs cycle (Brouillet et al. 2005). This induces a reduction in ATP production and promotes reactive oxygen species (ROS) production that leads to mitochondrial dysfunction and subsequent neuronal apoptosis (Kumar et al. 2011). The 3-NP model serves as a beneficial tool to evaluate the effect of novel treatments for HD.

Recombinant human erythropoietin (rhEPO) is a 165-amino acid glycoprotein that is widely used for treatment of different types of anemia (Fisher 2003). rhEPO has emerged as a neuroprotective agent against numerous types of central nervous system (CNS) insults. A study by Zhao et al. (2011) showed an anti-apoptotic effect of rhEPO after traumatic brain injury of rats. Furthermore, rhEPO treatment significantly prevented the streptozotocin-induced memory deficit by attenuating the hippocampal neuronal loss, neuroinflammation and cholinergic deficit in rats (Cevik et al. 2017). Previous studies indicate that rhEPO crosses the BBB in both humans and animals (Brines et al. 2000; Brines and Cerami 2005). Erythropoietin (EPO) and its receptors are expressed in the CNS by numerous kinds of cell types, such as astrocytes, neurons and endothelial cells (Mazur et al. 2010; Mallet et al. 2017), and could be up-regulated under distress and injury (Ott et al. 2015). Binding of EPO to its receptor induces a conformational change, initiating EPOR-associated janus kinase 2 (JAK2) tyrosine phosphorylation and multiple downstream signaling cascades including signal transducers and activators of transcription (STATs) and nuclear factor kappa B (NF-κB) (Parganas et al. 1998; Ott et al. 2015). Activation of these signaling cascades leads to up-regulation of anti-apoptotic and neuroprotective genes (Digicaylioglu and Lipton 2001; Zhao et al. 2011).

Interferon beta (IFN-β) is a member of the type I interferon family that is used as the primary treatment for multiple sclerosis (MS) (Kavrochorianou et al. 2016). In addition to immunomodulatory effects, IFN-β elicits a wide range of anti-inflammatory effects in the CNS (Dąbrowska et al. 2017; Mudò et al. 2019). IFN-β exerts its biological activity through the activation of the JAK/STAT signaling pathway (Haji Abdolvahab et al. 2016; Hurtado-Guerrero et al. 2017). Interferon type I receptor (IFNAR) activation by IFN-β induces STAT3 phosphorylation and subsequently inhibits inflammation and apoptosis (Dixon et al. 2016; Bolívar et al. 2018).

Respecting to these findings, the aim of the current study was to demonstrate the neuroprotective effect of rhEPO and IFN-β-1b in 3-NP-induced neurotoxicity and to investigate the potential underlying mechanisms. rhEPO or IFN-β in our study was given according to two different treatment regimens: either starting at the day of disease onset to reflect the clinical situation or at the day of first 3-NP injection as a prophylactic treatment.

## Materials and methods

### Animals

Adult male Wistar rats, weighing 200–250 g were purchased from Theodor Bilharz Research Institute, Cairo, Egypt. Animals were housed under controlled environmental conditions at constant temperature (25 ± 2 °C), humidity (60–70%), ventilation (10–20 changes/h) and a 12/12 h light/dark cycle with food and water ad libitum. The protocol used in this study complies with the Guide for Care and Use of Laboratory Animals published by the US National Institutes of Health (NIH Publication No. 85–23, revised 2011) and was approved by the Ethics Committee for Animal Experimentation at Faculty of Pharmacy, Cairo University (Permit Number: PT 1307).

### Drugs and chemicals

3-NP purchased from Sigma-Aldrich Chemical Co. (St. Louis, MO, USA) was dissolved in normal saline (0.9% w/v) in a volume of 0.5 mL/100 g animal body weight for intraperitoneal (i.p.) injection. rhEPO (Sedico Pharmaceutical Co., 6 October City, Egypt) and IFN-β-1b (Merck Serono Co., Ltd., Italy) were obtained from central administration of Biological and Innovative products and clinical studies. Fine chemicals and reagents were obtained from Sigma-Aldrich Chemical Co., unless indicated otherwise.

### Experimental design

**Fig. 1:**
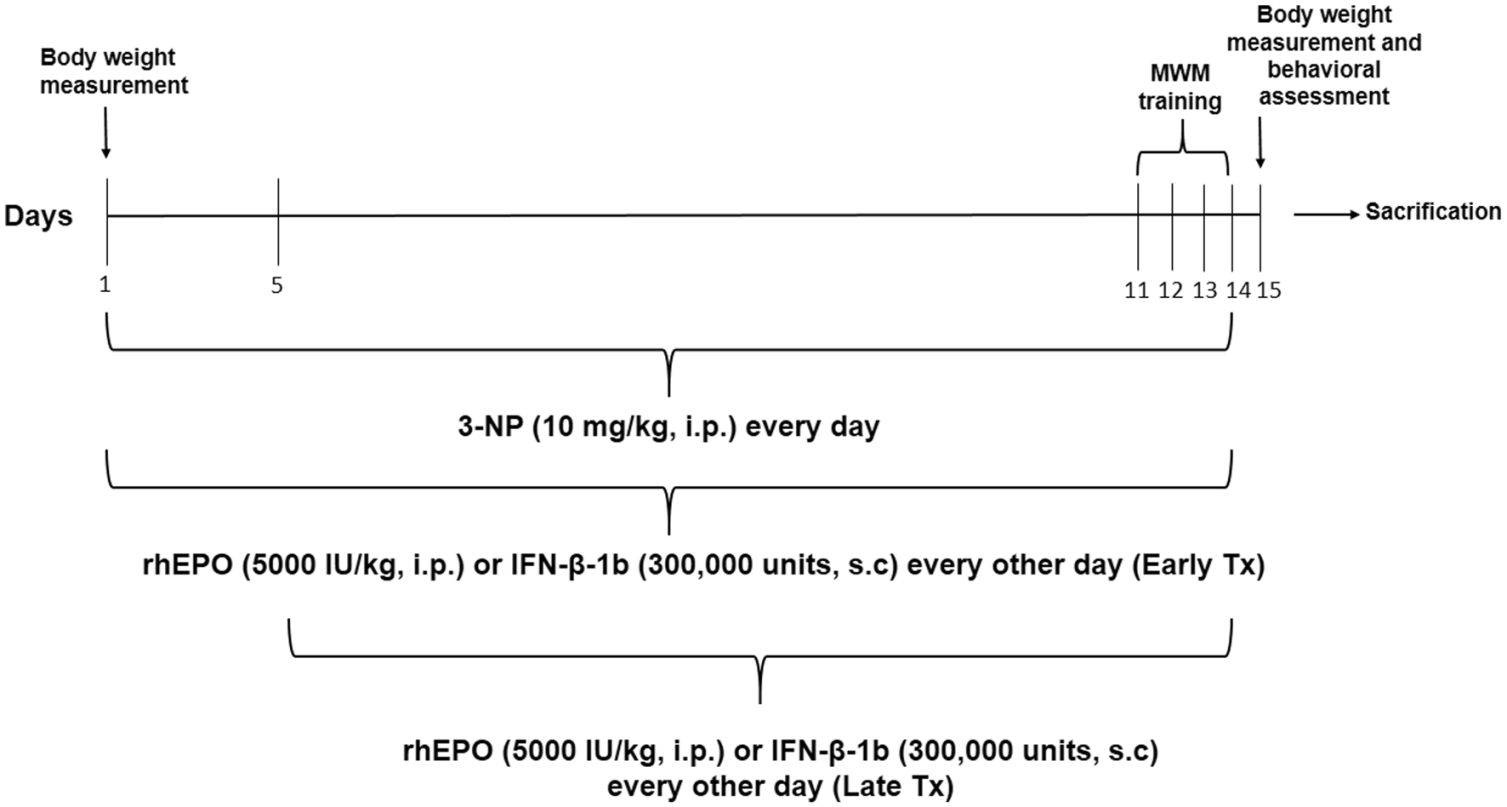
Schematic of experimental design. *3-NP* 3-nitropropionic acid, *rhEPO* recombinant human erythropoietin and, *INF-β-1b* interferon-beta-1b, *MWM* Morris water maze

As shown in Fig. 1, 60 rats were randomly divided into six groups (10 animals each). Group I (Control): received i.p. injection of normal saline and served as control group. Group II (3-NP): received 3-NP (10 mg/kg/day, i.p.) for 14 days (Kumar et al. 2011). Group III (rhEPO Early Tx): received 3-NP as in group II and rhEPO (5000 IU/kg/every other day, i.p) (Hamidi et al. 2013) for 14 days. Group IV (rhEPO Late Tx): rats received 3-NP as in group II and rhEPO (5000 IU/kg/every other day, i.p) starting from the 5th day of 3-NP injection for 10 days. Group V (IFN-β Early Tx): received 3-NP as in group II and IFN-β-1b (300,000 units, every other day, s.c.) (Dąbrowska et al. 2017) for 14 days. Group VI (IFN-β Late Tx): received 3-NP as in group II and IFN-β-1b (300,000 units, every other day, s.c.) starting from the 5th day of 3-NP injection for 10 days. rhEPO and IFN-β-1b were administrated 1 h before 3-NP injection. This regimen was based on the results of previous studies that revealed significant HD manifestations after the 5th injection of 3-NP (Maier et al. 2006; Malik et al. 2015).

### Measurement of body weight

On the 1st day and 24 h after the end of treatment, the body weight of animals was recorded to notice the change (Kumar et al. 2011).

### Behavioral assessment

Twenty-four hours after the last 3-NP injection, rats were subjected to behavioral tests; the open field, rotarod, and Morris water maze (MWM) tests were arranged in sequence from the least stressful test to the most stressful test with a 2 h rest period between the tests. All testing was conducted during the animal’s light cycle.

#### Open field test

Open field test was performed to monitor the spontaneous locomotor activity and exploratory behavior of rats. The apparatus was made of a square wooden box 80 × 80 × 40 cm in size. The walls were painted red and the floor was divided with white lines into 16 squares (4 × 4). Each rat was placed in the central area of the open field, and the locomotor activity was video recorded for 5 min. The latency time (time taken to leave the starting point), ambulation frequency (the number of squares crossed over by the rats) and rearing frequency (the frequency at which the rat stands on its hind limbs) were recorded for each animal. The test was performed in a quiet room under dim white light and the box was cleaned with 10% isopropyl alcohol and carefully dried to eliminate odors from the previous rat (Cummins and Walsh 1976).

#### Rotarod test

Rotarod test was used to evaluate the motor coordination of rats. The apparatus consisted of a rotating rod (120 cm in length, 3 cm in diameter and 30 cm in height) rotating at a constant speed of 25 rpm. Each rat performed three trials of 5 min each for 3 days with a 5 min gap between each trial to acclimatize to rotarod performance before starting the experiment. The latency time to fall off the rotarod was recorded as the time on rotarod (Dunham and Miya, 1957).

#### Morris water maze

MWM was used to assess spatial learning and memory in rats. The maze consisted of a stainless-steel circular pool (180 cm in diameter, 60 cm high) filled with water (28 ± 2 °C) to a depth of 35 cm and divided into four quadrants. A circular platform (9 cm in diameter) was placed in the center of one of the four quadrants, 1 cm below the water surface as an escape area. The rats received training sessions four times a day for four consecutive days. In all four trials, different positions were set as the starting point. The mean escape latency was recorded up to 120 s as maximum to climb the platform. On the 15th day of the experiment, the platform was removed and a dye was added to make the water opaque, then the animal was released randomly at any one of the edges to test the retention of the memory. The time taken to reach the quadrant where the platform was earlier placed was recorded (Morris 1984).

#### Brain processing

Twenty-four hours after behavioral testing, rats were killed by cervical dislocation under light anesthesia; subsequently, brains were rapidly excised and rinsed with ice-cold saline. For each group, two sets of rats were designated: one for biochemical investigations (*n* = 7) and the other (*n* = 3) for histological examinations. In the first set of samples (*n* = 7), striata were promptly dissected and stored at − 80 °C. One striatum was homogenized in ice-cold saline to prepare a 10% homogenate for the assessment of lipid peroxidation, reduced glutathione (GSH), complex II, III, and IV activity, tumor necrosis factor alpha (TNF-α), interleukin (IL)-6, NF-κB p65, brain-derived neurotrophic factor (BDNF), and caspase-3. The other striatum was used for RT-PCR and Western blot analyses. The protein contents of the tissue homogenates were determined using the Bradford (1976) method.

### Biochemical measurements

#### Determination of oxidative stress biomarkers

Oxidative stress status was estimated by the colorimetric determination of thiobarbituric acid reactive substances (TBARS) as lipid peroxidation end products and GSH according to the methods of Ohkawa et al. (1979) and Beutler et al. (1963), respectively. The results are expressed as nmol/mg protein for TBARS and mmol/mg protein for GSH.

#### Measurement of mitochondrial respiratory function

Complex I (NADH dehydrogenase) and IV (cytochrome oxidase) activities were determined by colorimetric assay (Biovision, Milpitas, CA, USA) according to the manufacturer’s protocol. Likewise, the enzyme activity of complex II (SDH) was determined by Microplate assay (Novagen, USA).

#### Enzyme-linked immunosorbent assay

Striatal TNF-α, NF-κB p65, and caspase-3 were estimated using rat ELISA kits (RayBiotech, Inc., Norcross, GA). Moreover, striatal IL-6 and BDNF were estimated using rat ELISA kits purchased from R and D system (Minneapolis, MN, USA) and MyBioSource Inc. (San Diego, CA, USA), respectively. The procedures were performed according to the manufacturer’s instructions. The results are expressed as pg/mg protein for TNF-α, IL-6, BDNF, and NF-κB p65 and ng/mg protein for caspase-3.

#### Western blot analysis

After protein extraction from striatal tissues using RIBA lysis buffer (Bio Basic Inc., Markham Ontario, L3R 8T4 Canada), equal amounts of protein were separated on a poly-acryl amide gel by SDS-PAGE using a Bio-Rad Mini-Protein II system. Polyvinylidene difluoride membranes was used to transfer the protein (Pierce, Rockford, IL, USA) with a Bio-Rad Trans-Blot Turbo system. Immunodetection of Western blots was conducted by incubating the membranes at room temperature for 1 h with blocking solution composed of 20 mM Tris–Cl (pH 7.5), 150 mM NaCl, 0.1% Tween 20 and 3% bovine serum albumin. Membranes were incubated overnight at 4 °C with one of the following primary antibodies (1:1000): p-(Tyr 1022/1023) JAK1 (Cat. no. 700028), p-(Tyr 1007–1008) JAK2 (Cat. no. PA5-85,735), p-(Tyr 705) STAT3 (Cat. no. 710093) and β-actin (Cat. no. MA5-15,739) obtained from Thermo Fisher Scientific Inc. (Rockford, IL). After washing, peroxidase-labeled secondary antibodies were added, and the membranes were incubated at 37 °C for 1 h. Analysis of the band intensity was performed using ChemiDoc™ imaging system with Image LabTM software version 5.1 (Bio-Rad Laboratories Inc., Hercules, CA, USA). The results are expressed as arbitrary units after normalization to β-actin protein expression.

#### Quantitative RT-PCR for Bax and Bcl-2

Total RNA was extracted from striatal tissues using an SV Total RNA Isolation system (Promega, Madison, WI, USA) and RNA purity was spectrophotometrically confirmed by optical density (OD) 260/280 nm. An RT-PCR (#K1621, Fermentas, Waltham, MA, USA) was used for extracted RNA reverse transcription into cDNA according to the manufacturer's instructions. To evaluate the mRNA expression of MBP, qRT-PCR was carried out using SYBR Green JumpStart Taq ReadyMix (Sigma–Aldrich, St. Louis, MO, USA) as described by the manufacturer. Briefly, 1 µg of total RNA was mixed with 50 µM. The primer sequences used are described in Table 1. PCR reaction included 5 min at 95 °C (activation) followed by 45 cycles at 95 °C for 5 s (denaturation) and at 60 °C for 10 s (annealing/extension). The relative expression of the target gene was obtained using the 2^−ΔΔCT^ formula (Pfaffl 2001). All values were normalized to that of β-actin which was used as the control and presented as fold change.

**Table 1:** Sequence of the primers used for RT-PCR

Reverse primer	Forward primer	Gene
5′-TAGGAGCCAGGGCAGTA-3′	5′-CGTTGACATCCGTAAAGAC-3′	β-actin
5′-AGCCACCCTGGTCTTG-3′	5′-GGTTGCCCTCTTCTACTTT-3′	Bax
5’-CGGTTCAGGTACTCAGCAT-3′	5′-ACTTTGCAGAGATGTCCAGT-3′	Bcl-2

### Histopathological examination

#### Assessment of striatal damage and Nissl staining

Brains were carefully removed from three rats per group, rinsed with ice-cold saline, and immediately fixed in 10% neutral buffered formalin for 72 h. Sagittal brain sections were processed for paraffin embedding and 3–5 μm sections were prepared using a rotatory microtome. Afterward, the sections were stained with hematoxylin and eosin (H and E) stains to examine the histological structure and cellular morphology of the striatum using light electric microscope.

Nissl staining was performed to demonstrate degenerated and intact neurons in the striatum. Sections were stained with toluidine blue stain for 3 min, air dried at room temperature for 1 h, and then shortly immersed in 70% alcohol. The average number of intact neurons was quantified from six random non-overlapping fields in different regions of the hippocampus in Nissl-stained tissue sections for each sample by using a Full HD microscopic camera operated by Leica Microsystems (GmbH, Wetzlar, Germany).

#### Glial fibrillary acidic protein (GFAP) immunostaining

Paraffin-embedded tissue sections of 3–5 μm thickness were first deparaffinized with xylene and then hydrated in serial dilutions of ethanol and heated in citrate buffer (pH 6.0) for 5 min. Next, the sections were blocked with 5% bovine serum albumin in phosphate buffered saline (PBS) for 60 min. The slides were then incubated in a dark chamber overnight at 4 °C with GFAP secondary antibody (Thermo Fisher Scientific Inc., Rockford, IL, USA). The slides were rinsed with PBS and incubated for 10 min in a solution of 0.02% diaminobenzidine (Dako, Copenhagen, Denmark). Sections were counterstained with Mayer's hematoxylin, dehydrated, cleared in xylene and then coverslipped for light microscopic examination. Six fields were randomly selected from each section, and positive signals within the section were highlighted, measured, and expressed as the percent area of expression of GFAP in the immunostained tissue sections using Leica Microsystems (GmbH, Wetzlar, Germany).

### Statistical analysis

The data obtained were expressed as means ± S.D. Data were analyzed using one-way ANOVA followed by the Tukey–Kramer multiple comparison test. Statistical analysis and graphical displaying were carried out using GraphPad Prism software version 7.04 (GraphPad Software Inc., CA, USA). The limit of significance was set and fixed at *p* < 0.05.

## Results

### Effect of rhEPO or IFN-β-1b on 3-NP-induced changes in body weight of rats

**Fig. 2:**
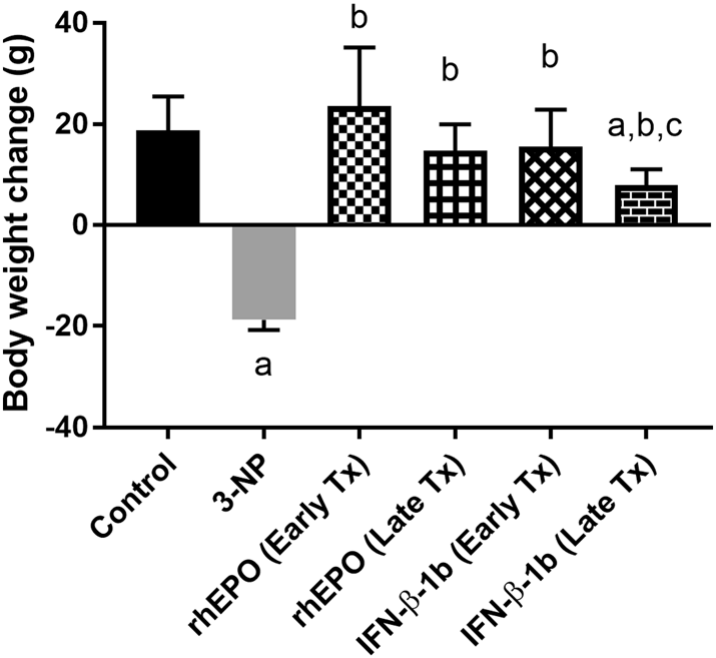
rhEPO and IFN-β-1b attenuate 3-NP-induced changes in body weight of rats. The results are the mean ± S.D. (*n* = 10). **a** Compared with the control group, **b** compared with the 3-NP group, and c compared with the rhEPO (Early Tx) group. All values are statistically significant at *p* < 0.05. *3-NP* 3-nitropropionic acid, *rhEPO* recombinant human erythropoietin and, *INF-β-1b* interferon-beta-1b

3-NP intoxication caused a significant decrease in body weight of rat as compared with the control group. On the other hand, early and late rhEPO or IFN-β-1b treatment significantly hindered body weight loss compared with the 3-NP group (Fig. 2).

### Effect of rhEPO and IFN-β-1b on 3-NP-induced changes in motor activity and coordination of rats

**Fig. 3:**
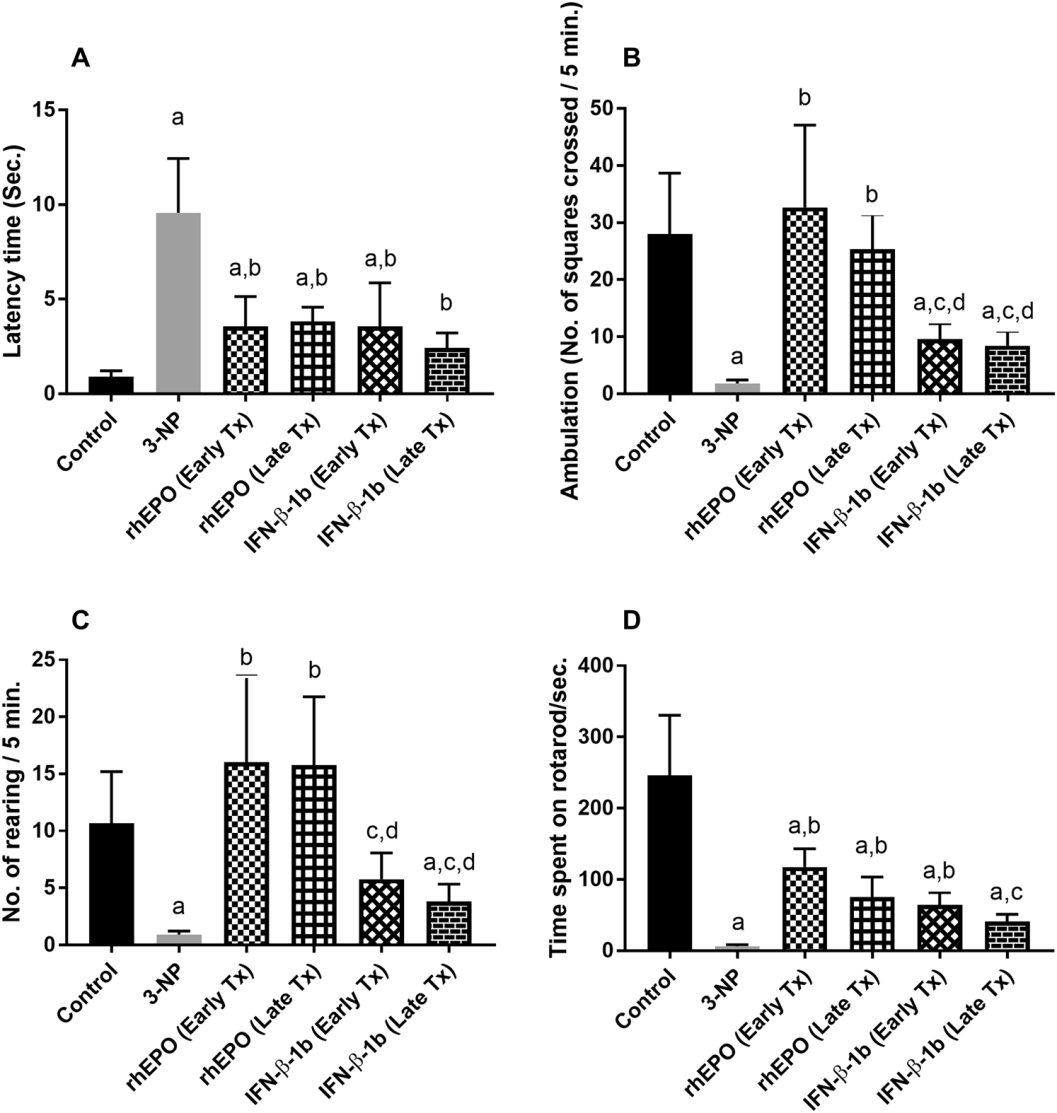
rhEPO and IFN-β-1b mitigate 3-NP-induced changes in motor activity and coordination of rats. **A** Latency time, **B** ambulation frequency, **C** rearing frequency, and **D** time on rotarod. The results are the mean ± S.D. (*n* = 10). **a** Compared with the control group, **b** compared with the 3-NP group, **c** compared with the rhEPO (Early Tx) group, and **d** compared with rhEPO (Late Tx) group. All values are statistically significant at *p* < 0.05. *3-NP* 3-nitropropionic acid, *rhEPO* recombinant human erythropoietin and, *INF-β-1b* interferon-beta-1b

3-NP produced a prominent deterioration in the motor performance and coordination of rats as evidenced by the open field and rotarod tests. In the open field test, 3-NP showed a marked increase in latency time to reach 10.6-fold, and a profound decrease in ambulation and rearing frequencies to reach 6.43 and 8.44%, respectively, as compared with the control group. In contrast, early and late rhEPO treatment produced a marked decrease in latency time and increase in ambulation and rearing frequencies as compared to the 3-NP group. However, early and late IFN-β-1b succeeded to significantly decrease latency time only, while ambulation and rearing frequencies were insignificantly effected as compared with the 3-NP group. Moreover, 3-NP showed a marked decrease in time spent on rotarod to reach 2.52% as compared with the control group. Treatment with early rhEPO, late rhEPO and early INF-β-1b markedly reversed the decrease in time spent on rotarod to reach 18.9-fold, 12.1-fold and 10.4-fold, respectively, as compared with the 3-NP group (Fig. 3). In contrast, late INF-β-1b did not significantly affect the time spent on rotarod.

### Effect of rhEPO and IFN-β-1b on 3-NP-induced changes in spatial and learning memory in rats

**Fig. 4:**
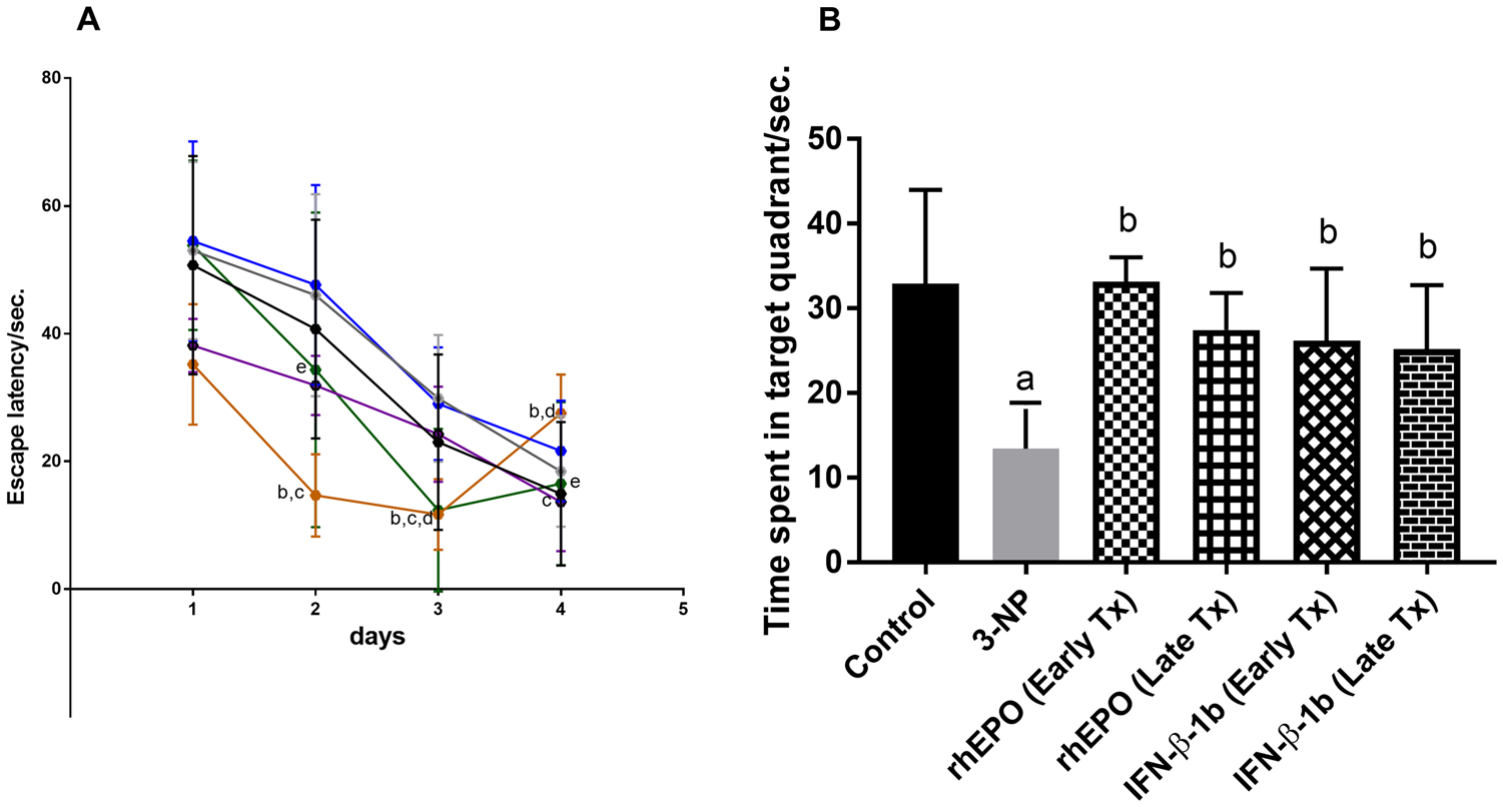
rhEPO and IFN-β-1b reverse 3-NP-induced changes in spatial and learning memory in rats. **A** MWM training and **B** MWM probe test. The results are the mean ± S.D. (*n* = 10). **a** Compared with the control group, **b** compared with the 3-NP group, **c** compared with the rhEPO (Early Tx) group, **d** compared with rhEPO (Late Tx) group, and **e** compared with INF-β-1b (Early Tx). All values are statistically significant at *p* < 0.05. *3-NP* 3-nitropropionic acid, *rhEPO* recombinant human erythropoietin and, *INF-β-1b* interferon-beta-1b, *MWM*: Morris water maze

The results of the MWM test revealed that 3-NP-treated rats showed no significant difference in escape latency in all days of training (Fig. 4A). In the probe test, 3-NP-treated rats failed to recall the exact location of the platform and spent less time in the target quadrant. However, all treatment groups of rhEPO and INF-β-1b reverted the time spent in target quadrant to its normal values (Fig. 4B).

### Effect of rhEPO and IFN-β-1b on 3-NP-induced changes in oxidative stress biomarkers

**Fig. 5:**
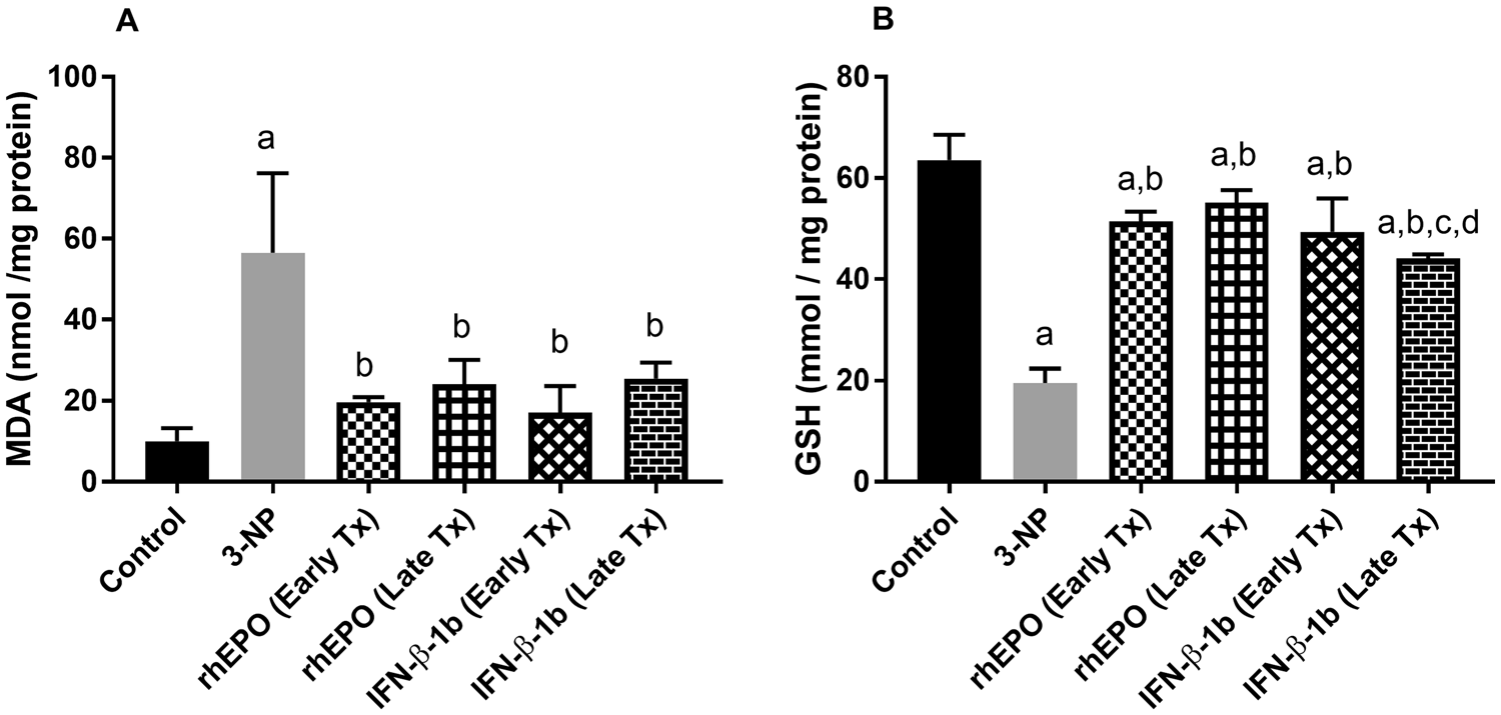
rhEPO and IFN-β-1b reduce 3-NP-induced oxidative stress. **A** Striatal MDA level and **B** striatal GSH level. The results are the mean ± S.D. (*n* = 7). **a** Compared with the control group, **b** compared with the 3-NP group, **c** compared with the rhEPO (Early Tx) group, and **d** compared with rhEPO (Late Tx) group. All values are statistically significant at *p* < 0.05. *3-NP* 3-nitropropionic acid, *rhEPO* recombinant human erythropoietin and, *INF-β-1b* interferon-beta-1b, *MDA* malondialdehyde, *GSH* glutathione

3-NP injection produced a significant increase in striatal MDA to reach 5.6-fold as compared with the control group. Treatment with early rhEPO, late rhEPO, early INF-β-1b, and late INF-β-1b markedly reversed the increase in MDA to reach 34.81, 42.71, 30.27 and 45.25%, respectively, as compared with 3-NP group values. On the other hand, 3-NP produced a significant decrease in striatal GSH to reach 30.73% as compared with the control group. However, treatment with early rhEPO, late rhEPO, early INF-β-1b, and late INF-β-1b increased GSH to reach 2.6-fold and 2.8-fold, 2.5-fold, and 2.3-fold, respectively, as compared with 3-NP group values (Fig. 5).

### Effect of rhEPO and IFN-β-1b on 3-NP-induced changes in mitochondrial enzyme complexes

**Fig. 6:**
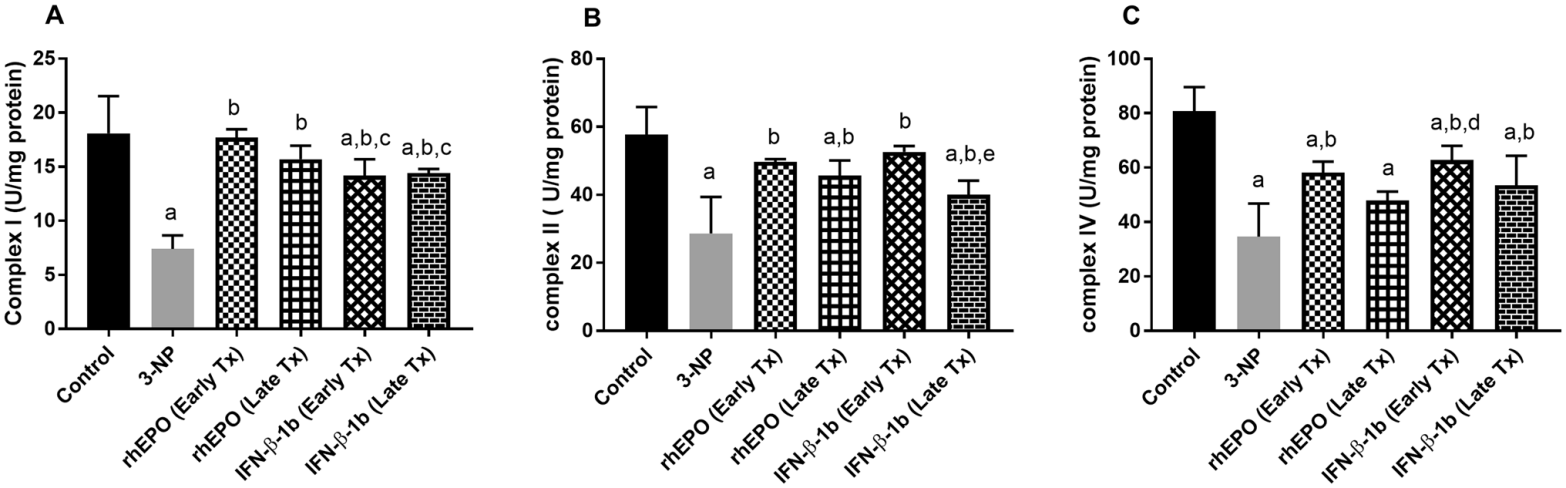
rhEPO and IFN-β-1b attenuate 3-NP-induced changes in mitochondrial enzyme complexes. **A** Complex I, **B** complex II, and **C** complex IV. The results are the mean ± S.D. (*n* = 7). **a** Compared with the control group, **b** compared with the 3-NP group, **c** compared with the rhEPO (Early Tx) group, **d** compared with rhEPO (Late Tx) group, and **e** compared with INF-β-1b (Early Tx). All values are statistically significant at *p* < 0.05. *3-NP* 3-nitropropionic acid, *rhEPO* recombinant human erythropoietin and, *INF-β-1b* interferon-beta-1b

3-NP-treated rats showed a significant decrease in the mitochondrial enzyme complexes I, II, and IV to reach 40.90, 49.62, and 42.84% as compared with the control group. On the other hand, treatment with early rhEPO, late rhEPO, early INF-β-1b, and late INF-β-1b succeeded in increasing the levels of complex I significantly to reach 2.4-fold, 2.1-fold, 1.9-fold, and twofold as compared with the 3-NP group values. In addition, treatment with early rhEPO, late rhEPO, early INF-β-1b, and late INF-β-1b markedly reversed the decrease in complex II to reach 1.7-fold, 1.6-fold, 1.8-fold, and 1.4-fold, respectively, as compared with the 3-NP-treated group. Similarly, treatment with early rhEPO, late INF-β-1b, and early INF-β-1b markedly increased the levels of complex IV to reach 1.7-fold, 1.8-fold, and 1.6-fold, respectively, as compared with 3-NP-treated rats (Fig. 6). We also observed that early INF-β-1b group showed significantly higher complex II activity than late INF-β-1b group.

### Effect of rhEPO and IFN-β-1b on 3-NP-induced changes in pro-inflammatory cytokines

**Fig. 7:**
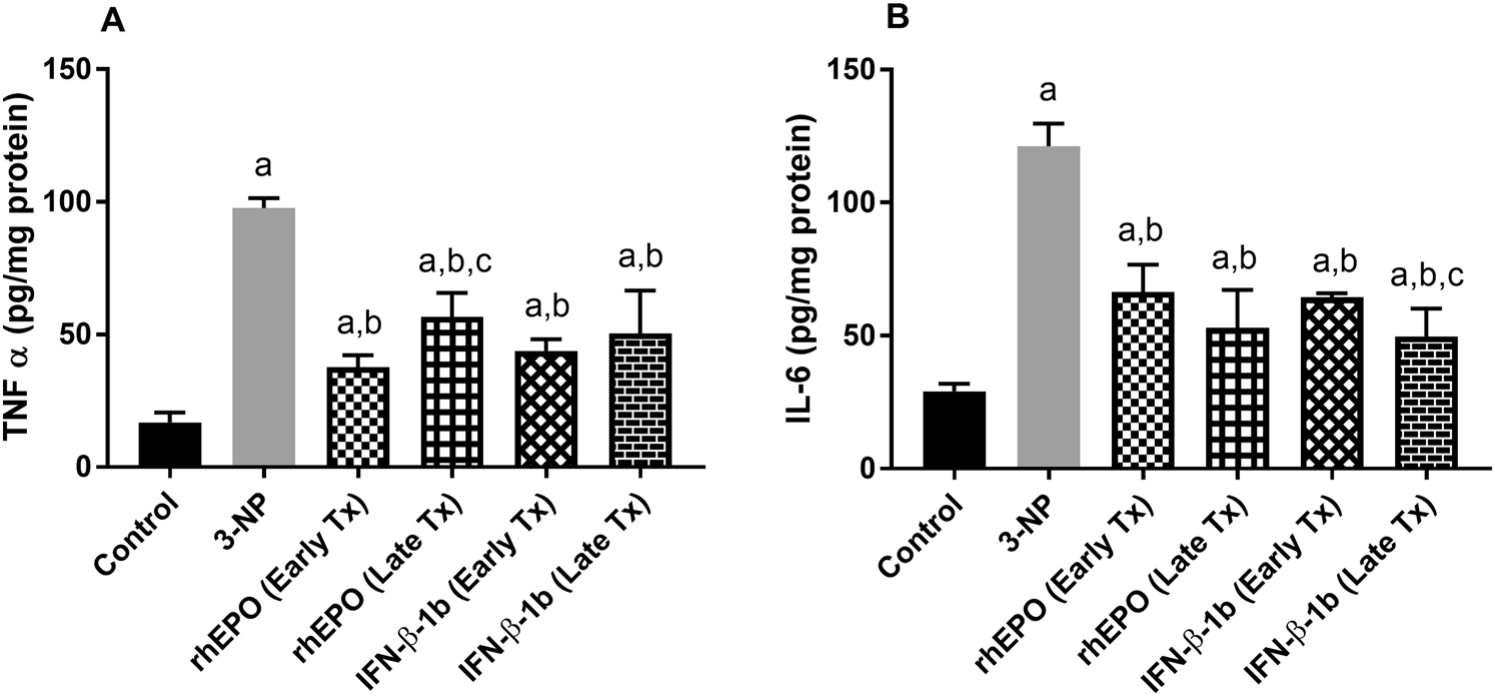
rhEPO and IFN-β-1b decline 3-NP-induced increases in pro-inflammatory cytokines. **A** Striatal TNF-α level and **B** striatal IL-6 level. The results are the mean ± S.D. (*n* = 7). **a** Compared with the control group, **b** compared with the 3-NP group, and **c** compared with the rhEPO (Early Tx) group. All values are statistically significant at *p* < 0.05. *3-NP* 3-nitropropionic acid, *rhEPO* recombinant human erythropoietin and, *INF-β-1b* interferon-beta-1b, *TNF-α* tumor necrosis factor alpha, *IL-6* interlukin-6

3-NP intoxication caused a significant increase in striatal TNF-α and IL-6 levels to reach 5.8-fold and 4.2-fold, respectively, as compared with the control group. Treatment with early rhEPO, late rhEPO, early INF-β-1b, and late INF-β-1b significantly attenuated the elevation in TNF-α level to reach 38.54, 58.02, 44.82, and 51.64%, respectively, as compared with the 3-NP-treated group. Moreover, treatment with early rhEPO, late rhEPO, early INF-β-1b, and late INF-β-1b showed a significant decrease in the levels of IL-6 to reach 54.80, 43.66, 53.12 and 40.97%, respectively, as compared with the 3-NP group values (Fig. 7).

### Effect of rhEPO and IFN-β-1b on 3-NP-induced changes in the JAK/STAT pathway

**Fig. 8:**
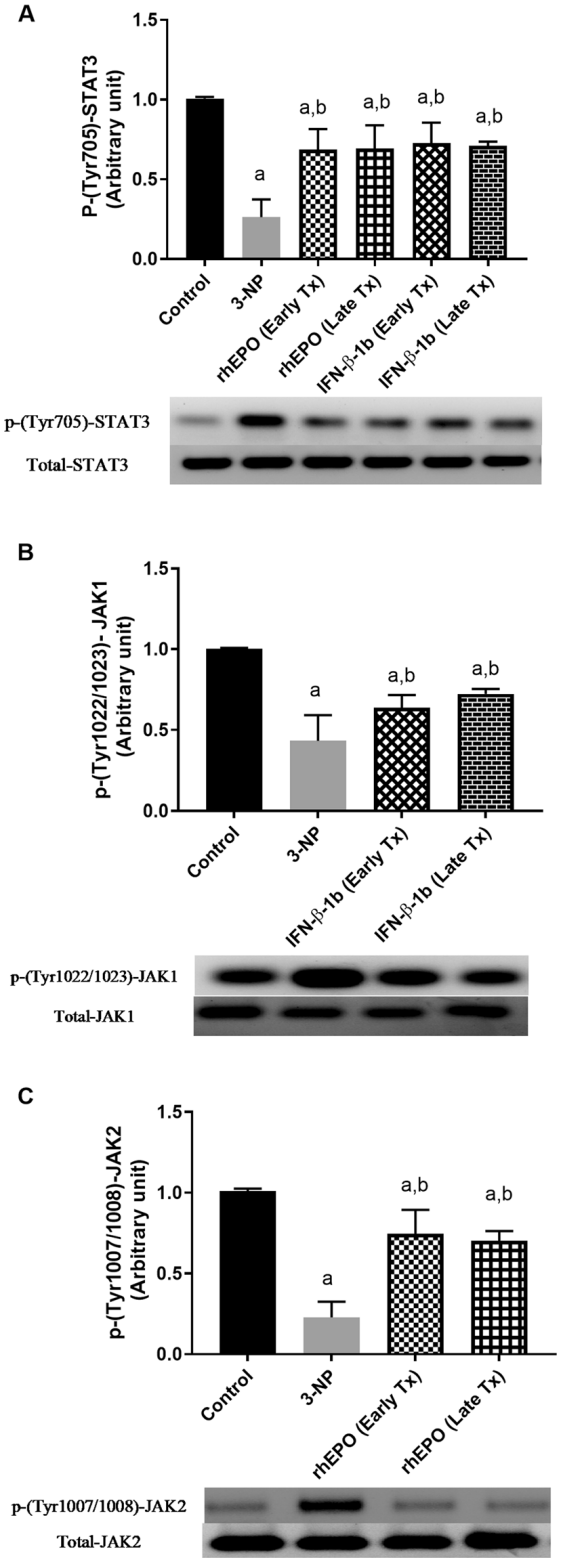
rhEPO and IFN-β-1b upregulate the JAK/STAT pathway. **A** p-(Tyr 705)-STAT3 expression, **B** p-(Tyr 1022/1023)-JAK1 expression, and **C** p-(Tyr 1007–1008)-JAK2 expression. The results are the mean ± S.D. (*n* = 7). **a** Compared with the control group and **b** compared with the 3-NP group. All values are statistically significant at *p* < 0.05. *3-NP* 3-nitropropionic acid, *rhEPO* recombinant human erythropoietin and, *INF-β-1b* interferon-beta-1b

3-NP injection down-regulated the expression of p-JAK1, p-JAK2, and p-STAT3 to reach 43.19, 22.55, and 26.08%, respectively, as compared with the control group. Conversely, treatment with early INF-β-1b and late INF-β-1b up-regulated the expression of p-JAK1 to reach 1.5-fold and 1.7-fold, respectively, as compared with the 3-NP-treated group. Similarly, treatment with early rhEPO and late rhEPO markedly improved the expression of p-JAK2 to reach 3.3-fold and 3.1-fold, respectively, as compared with the 3-NP-treated group values. Treatment with early rhEPO, late rhEPO, early INF-β-1b, and late INF-β-1b alleviated the decreases in p-STAT3 to reach 2.6-fold, 2.6-fold, 2.8-fold, and 2.7-fold, respectively, as compared with the 3-NP group (Fig. 8).

### Effect of rhEPO and IFN-β-1b on 3-NP-induced changes in BDNF and NF-κB p65

**Fig. 9:**
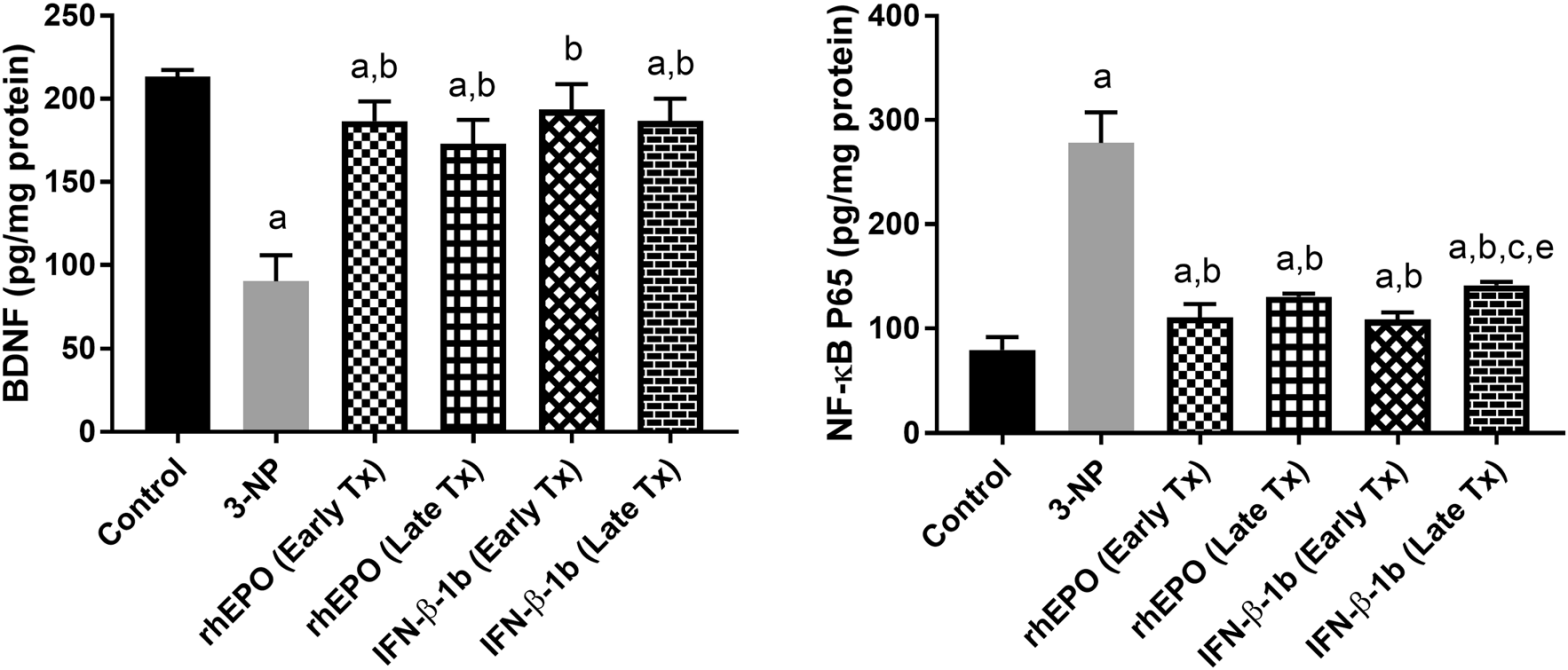
Effect of rhEPO and IFN-β-1b on 3-NP-induced changes in **A** BDNF and **B** NF-κB p65. The results are the mean ± S.D. (*n* = 7). **a** Compared with the control group, **b** compared with the 3-NP group, **c** compared with the rhEPO (Early Tx) group, and **e** compared with IFN-1b (Early Tx) group. All values are statistically significant at *p* < 0.05. *3-NP* 3-nitropropionic acid, *rhEPO* recombinant human erythropoietin and, *INF-β-1b* interferon- beta-1b

A substantial decrease in the level of BDNF to reach 3.5-fold with a concomitant increase in NF-κB p65 level to reach 42.34% was observed in the striatum of 3NP-treated rats compared to that in control rats. Treatment with early rhEPO, late rhEPO, early INF-β-1b, and late INF-β-1b increased BDNF level to reach 2.1-fold, 1.9-fold, 2.1-fold, and 2.1-fold, respectively, as compared with the 3-NP-treated group. Furthermore, early rhEPO, late rhEPO, early INF-β-1b, and late INF-β-1b decreased NF-κB p65 level to reach 39.83, 46.89, 39.10, and 50.92%, respectively, as compared with the 3-NP-treated group (Fig. 9). A statistically significant benefit in early INF-β-1b treatment on decreasing NF-κB p65 level was observed compared with the late INF-β-1b group.

### Effect of rhEPO and IFN-β-1b on 3-NP-induced changes in apoptotic biomarkers

**Fig. 10:**
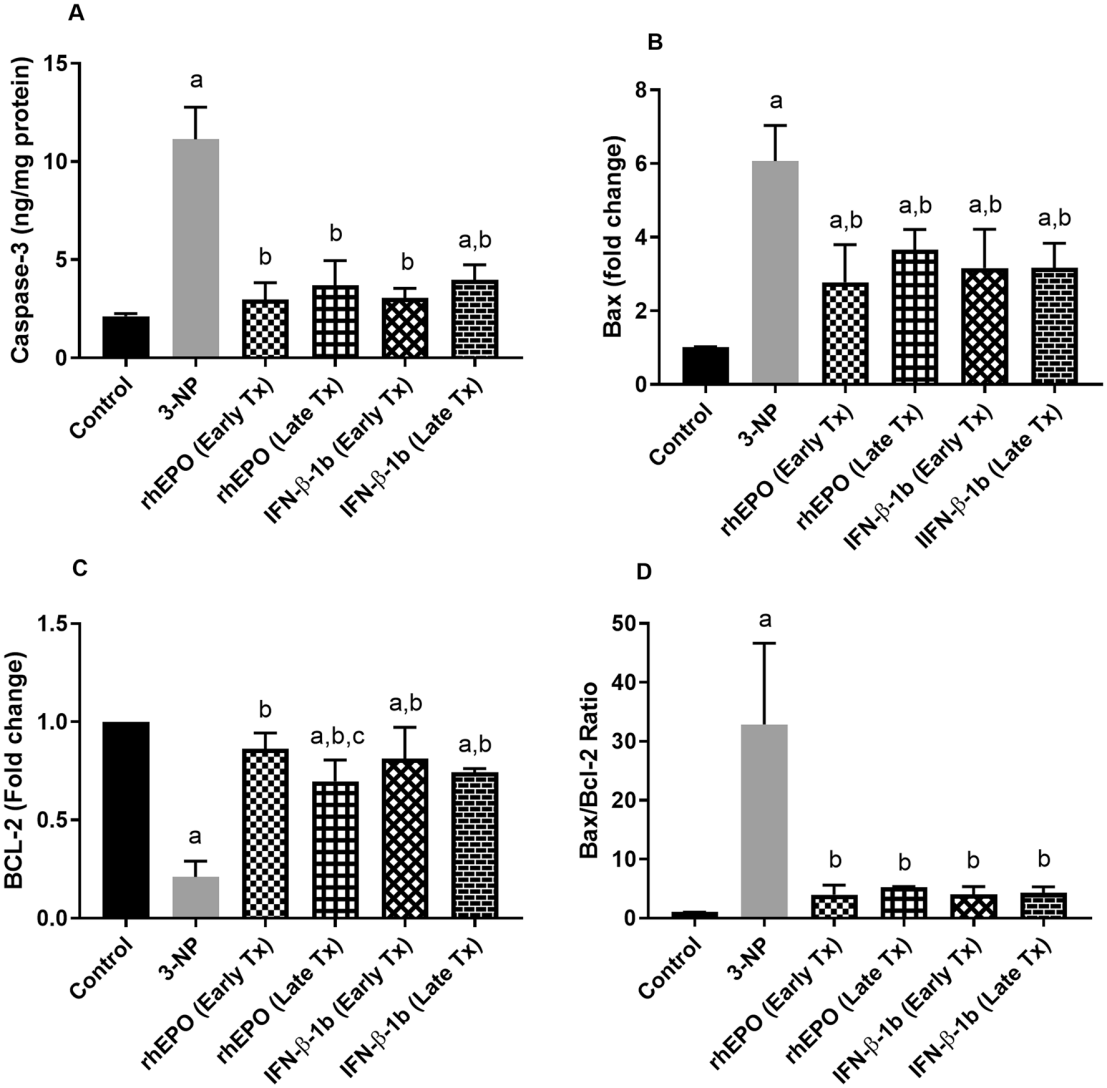
Effect of rhEPO and IFN-β-1b on 3-NP-induced changes in **A** Caspase-3, **B** Bax, **C** Bcl-2, and **D** Bax/Bcl-2 ratio. The results are the mean ± S.D. (*n* = 7). a Compared with the control group, **b** compared with the 3-NP group, and **c** compared with the rhEPO (Early Tx) group. All values are statistically significant at *p* < 0.05. *3-NP* 3-nitropropionic acid, *rhEPO* recombinant human erythropoietin and, *INF-β-1b* interferon-beta-1b

3-NP intoxication caused an elevation in striatal caspase-3 level, mRNA expression of Bax, and Bax/Bcl2 ratio to reach 5.2-fold, 6.1-fold, and 32.5-fold, respectively, as compared with the control group. Besides, 3-NP produced a significant decrease in Bcl2 mRNA level to reach 21% as compared with the control group. However, treatment with early rhEPO, late rhEPO, early INF-β-1b, and late INF-β-1b succeeded to reverse the increase in caspase-3 to reach 26.68, 33.17, 27.46, and 35.63%, respectively, as compared with the 3-NP-treated group. Treatment with early rhEPO, late rhEPO, early INF-β-1b, and late INF-β-1b significantly decreased Bax mRNA levels to reach 45.68, 60.21, 52.14, and 52.20%, respectively, as compared with the 3-NP group values. Treatment with early rhEPO, late rhEPO, early INF-β-1b, and late INF-β-1b markedly decreased Bax/Bcl-2 to reach 12.12, 16, 12.19, and 13.06%, respectively, as compared with 3-NP group. Treatment with early rhEPO, late rhEPO, early INF-β-1b, and late INF-β-1b significantly increased Bcl2 mRNA levels to reach 4.1-fold, 3.3-fold, 3.9-fold, and 3.5-fold, respectively, as compared with the 3-NP-treated group (Fig. 10).

### Effect of rhEPO and IFN-β-1b on 3-NP-induced histopathological changes

**Fig. 11:**
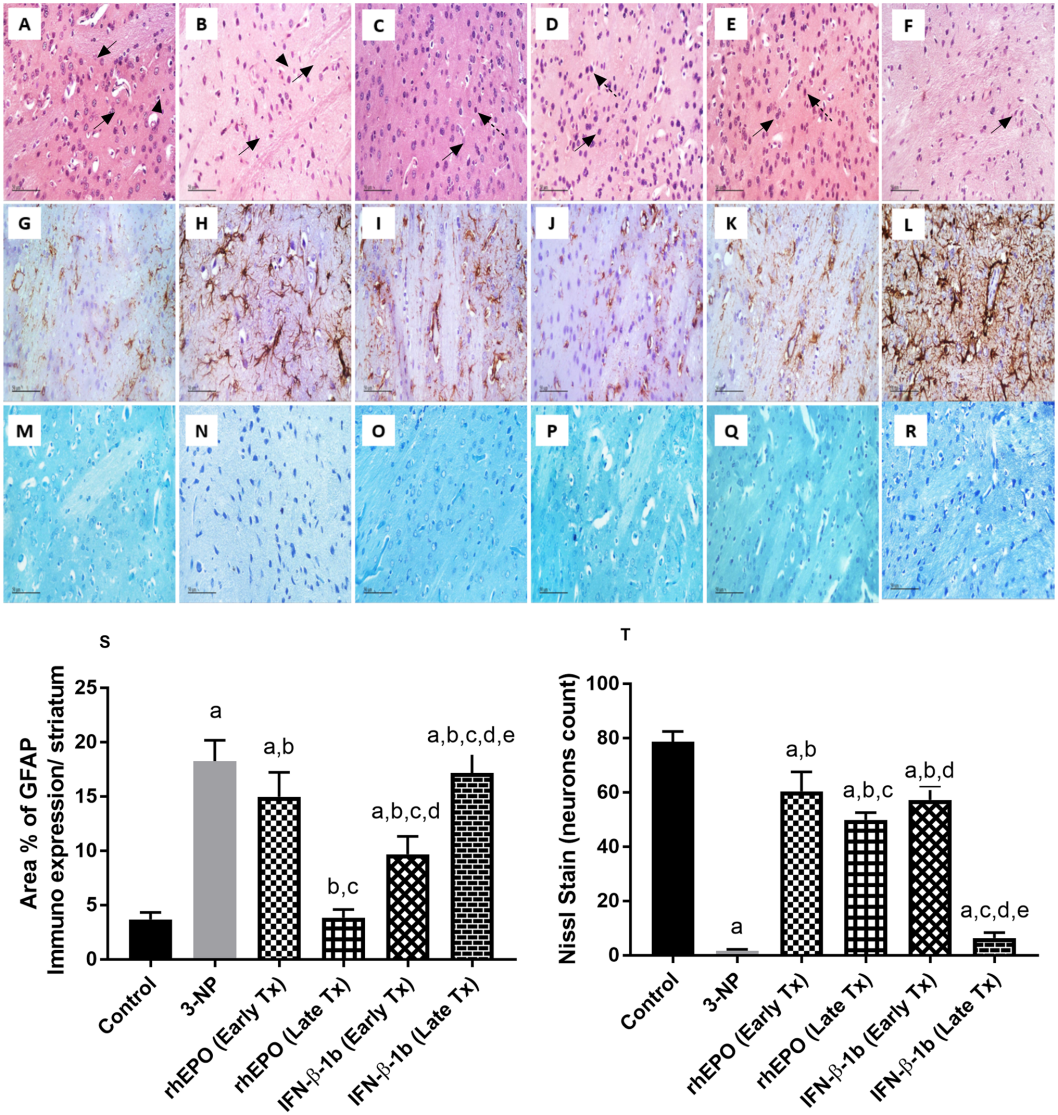
Effect of rhEPO and IFN-β-1b on 3-NP-induced histopathological changes. (**A**-**F**) Specimen stained with (**H**-**E**) (400 × magnification). **A** Control group demonstrated normal histological structures of striatum including many intact neurons with characteristic large vesicular nuclei with prominent nucleoli (arrows), intact intercellular tissue and few scattered glial cells (arrowhead). **B** 3-NP group showed extensive damaged shrunken degenerated and apoptotic neurons losing its cellular details (arrows) with mild perineuronal edema as well as moderate activation of glial cells (arrowhead). **C** rhEPO (Early Tx), **D** rhEPO (Late Tx), and **E** IFN-β-1b (Early Tx) groups revealed many apparent intact striatal neurons (arrow) with occasionally few scattered degenerated pyknotic cells (dashed arrow) as well as mild glial cells infiltration. **F** IFN-β-1b (Late Tx) showed moderate neuronal and degeneration (arrow) as well as mild glial infiltration. (**G**-**L**) Specimens stained with GFAP (400 × magnification). **G** The control group showed normal GFAP immunostaining of inactivated astrocytes. **H** 3-NP group showed an obvious increase in GFAP-immunoreactive astrocytes. **I** rhEPO (Early Tx) group showed moderate increase in GFAP-immunoreactive astrocytes. **J** rhEPO (Late Tx) group showed mild increase in GFAP-immunoreactive astrocytes. **K** IFN-β-1b (Early Tx) group showed mild increase in GFAP-immunoreactive astrocytes. **L** IFN-β-1b (Late Tx) showed moderate increase in GFAP-immunoreactive astrocytes. (**M**–**R**) Specimens stained with Nissl of 400 × magnification. **S** % area of GFAP-immunoreactive astrocytes. **T** Number of Nissl stain (intact neurons count). The results are the mean ± S.D. (*n* = 7). **a** Compared with the control group, **b** compared with the 3-NP group, **c** compared with rhEPO (Early Tx) group, **d** compared with rhEPO (Late Tx) group, and **e** compared with IFN-β-1b (Early Tx) group. All values are statistically significant at *p* < 0.05. *3-NP* 3-nitropropionic acid, *rhEPO* recombinant human erythropoietin and, *INF-β-1b* interferon-beta-1b

Microscopic examination showed that control group demonstrated normal histological structures of striatum. However, the 3-NP group showed extensive damaged shrunken degenerated and apoptotic neurons losing its cellular details with mild perineuronal edema as well as moderate activation of glial cells. Sections from early rhEPO, late rhEPO, and early IFN-β-1b treatment groups revealed many apparent intact striatal neurons with occasionally few scattered degenerated pyknotic cells as well as mild glial cells infiltration. However, sections from late IFN-β-1b showed moderate neuronal loss and degeneration as well as mild glial cell infiltration (Fig. 11A-F).

3-NP produced a significant increase in GFAP immunoreactivity to reach fivefold as compared with the control group. On the other hand, early rhEPO, late rhEPO, early IFN-β-1b and late IFN-β-1b showed a marked decrease in GFAP immunoreactivity to reach 82, 21.10, 52.97, and 93.99%, respectively, as compared with the 3-NP group. A statistically significant benefit in early INF-β-1b treatment on decreasing GFAP immunoreactivity was observed compared with the late INF-β-1b group (Fig. 11G-M).

3-NP group showed a significant decrease in the number of intact viable cells in Nissl staining to reach 2.12% as compared with the control group. However, treatment with early rhEPO, late rhEPO, and early IFN-β-1b remarkably reverse this decrease to reach 36.20, 29.90, and 36.20-fold, respectively, as compared with 3-NP-treated group. In contrast, late IFN-β-1b showed a non-significant change of the intact number of viable cells compared to 3-NP group. Early INF-β-1b treatment group showed a significant benefit in reversing the decrease in the intact number of viable cells comparing with the late INFβ-1b group (Fig. 11N-R).

## Discussion

The present study provides the first evidence for the neuroprotective effect of rhEPO and IFN-β-1b in 3-NP-induced neurotoxicity. This notion is supported by: (1) improved motor and cognitive impairments; (2) restored mitochondrial complex enzyme activities; (3) alleviation of striatal oxidative stress, inflammation, and neuronal death; and (d) activation of JAK/STAT pathway. It is also apparent that both early and late treatments of rhEPO or IFN-β-1b showed benefit on HD-like symptoms.

Patients with HD experience motor, cognitive, and psychiatric symptoms (Purdon et al. 1994). The characteristic motor symptoms are chorea, hypokinesia, ataxia, and dystonia (Snowden 2017). In the present study, the systemic administration of 3-NP produces significant motor impairments, loss of grip strength, and cognitive dysfunction as evidenced by open field, rotarod, and MWM tests, which mimic the late phase symptoms of HD. The present results are in agreement with the previous reports, indicating motor and cognitive impairment following the administration of 3-NP which was associated with the hippocampal CA1 and CA3 neuronal injury (Danduga et al. 2018; Sayed et al. 2020). However, treatment with rhEPO or IFN-β-1b significantly reversed these motor and cognitive impairments. In parallel, previous studies showed that rhEPO attenuates Parkinson’s disease-induced motor deficits (Thompson et al. 2020) and protects against cognitive impairment (Sayed et al. 2019) in mice. Likewise, IFN-β is reported to ameliorate cognitive dysfunction in rat models of Alzheimer’s disease (Chavoshinezhad et al. 2019a, b).

In the present study, the injection of 3-NP produces a significant reduction in body weights of the animals. 3-NP-induced loss in body weight might be due to metabolic dysfunction (Danduga et al. 2018). In addition, striatal lesions and hypokinesia could be the possible reason behind anorexia and body weight reduction (Keene et al. 2001). However, treatment with rhEPO or IFN-β-1b mitigated 3-NP-induced body weight loss due to attenuation of 3-NP-induced neurotoxicity.

The JAK/STAT signaling pathway has an essential role in the regulation of immune responses (Schindler et al. 2007). The dysregulation of this pathway has pathological implications in various neurodegenerative and neuroinflammatory disorders (Träger et al. 2013). The activation and cross-phosphorylation of JAK tyrosine kinases occurs when cytokines bind to their receptors. STATs are subsequently phosphorylated by JAK and translocate into nucleus to induce transcription of target genes (Träger et al. 2013). Numerous biochemical studies have revealed that JAK1 is involved in signaling by members of class II cytokine receptors including type I IFN-α/β, while JAK2 is implicated in signaling by members of single chain receptors such as EPOR (Zhao et al. 2011; Dixon et al. 2016; Wei et al. 2017). Based on this theory, we assumed that JAK/STAT pathway may participate in the neuroprotective activity of rhEPO and IFN-β-1b against 3-NP-induced neurotoxicity. In the present study, we found that 3-NP intoxication down-regulated JAK1, JAK2, and STAT3 protein expression in striatum of rat. However, our data shows that rhEPO administration up-regulated the JAK2/STAT3 signaling pathway, while IFN-β-1b administration up-regulated JAK1/STAT3 signaling in the striatum of 3-NP-treated rats. Hereafter, the study revealed that the activation of JAK/STAT signaling by rhEPO and IFN-β-1b suppressed mitochondrial dysfunction, oxidative stress, neuroinflammation, and apoptosis, the key pathological hallmarks of HD.

Mitochondrial dysfunction leading to disturbed energy production has a crucial role in HD. 3-NP reversibly inhibits complex II (SDH) that prevents electrons from being transferred from complex II to complex III, resulting in decreased ATP levels and metabolic disruption leading to excitotoxic cell death (Scallet et al., 2001). Herein, animals treated with 3-NP show inhibition of the activities of mitochondrial complexes I, II, and IV. In agreement, previous studies reported that 3-NP causes impairment in mitochondrial enzyme complex activities in rats (Kumar and Kumar 2009; Kumar et al. 2011). However, treatment with rhEPO or IFN-β-1b significantly restored mitochondrial enzymes activities. It is worth mentioning that early INF-β-1b significantly increased complex II activity as compared to late INF-β-1b, which might be due to the specificity of 3-NP toward complex II.

3-NP-induced mitochondrial dysfunction and inhibition of energy production increase electrons released from mitochondria and generation of ROS (Gil and Rego 2008). Our data shows that 3-NP administration induced oxidative stress in striatum of rats as evident by increase in MDA level and decrease in GSH content. rhEPO or IFN-β-1b treatment reduced MDA level and restored GSH content in the striatum, suggesting their anti-oxidant-like properties. Similar to our data, previous studies reported the anti-oxidant activity of rhEPO and IFN-β in neurodegenerative diseases (Mudò et al. 2019; Thompson et al. 2020).

In addition to oxidative stress, neuroinflammation is a well-established feature of HD-neuropathology. Astrocytes exposed to an inflammatory stimulus produce pro-inflammatory cytokines, such as TNF-α and IL-6, which directly activate NF-κB p65 triggering inflammatory and apoptotic pathways, resulting in loss of neuronal activity (Neal and Richardson 2018). In parallel, our results showed increased striatal TNF-α, IL-6, and NF-κB p65 levels in 3-NP-treated rats. However, rhEPO or IFN-β-1b signified their anti-inflammatory effects by abrogating the striatal levels of TNF-α, IL-6, and NF-κB p65 as compared to the 3-NP-treated group. Likewise, the anti-inflammatory effect of rhEPO or IFN-β-1b against neuronal inflammation was previously reported (Sengul et al. 2013; Wei et al. 2017). Herein, 3-NP injection also potentiated striatal apoptotic cascade as indicated by increased expression of the pro-apoptotic protein Bax as well as level of caspase-3 and decreased expression of the anti-apoptotic protein Bcl2. This can be attributed to the inhibition of SDH by 3-NP which causes mitochondrial dysfunction and activation of apoptotic cascade (Sandhir et al. 2010). On the other hand, treatment with rhEPO or IFN-β-1b modulated the expression of apoptotic proteins in the striatum showing a significant anti-apoptotic effects. These findings are supported by previous studies which showed the potent anti-apoptotic activity of rhEPO or IFN-β (Pillai et al. 2008; Chavoshinezhad et al. 2019a).

Our findings were correlated with the histopathological changes evidenced by the increased striatal injury score and GFAP immunoreactivity as well as extensive neuronal loss shown by Nissl staining indicating neurodegeneration and reactive changes after 3-NP exposure. The elevation of GFAP following astrogliosis has been observed as a long-standing pathological feature in 3-NP model of HD (Ramachandran and Thangarajan 2018; Elbaz et al. 2019). However, treatment with rhEPO or IFN-β-1b reversed these histopathological changes evidencing their neuroprotective properties. Interestingly, a statistically significant benefit in early INF-β-1b treatment on decreasing NF-κB p65 level and GFAP immunoreactivity was observed compared with the late INF-β-1b group. Previous study by Kappos et al. (2007) suggested that early initiation of treatment with IFN-β-1b prevents the development of disability, supporting its use after the first manifestation of relapsing–remitting multiple sclerosis.

Excessive release of pro-inflammatory cytokines can lead to reduced production of neurotrophins (Yirmiya and Goshen 2011). BDNF is a well-known neurotrophic factor that plays a crucial role in maintaining neuronal survival and differentiation (Zuccato and Cattaneo 2007). Post-mortem examination has revealed reduced expression of BDNF in the striatum of HD brains (Ferrer et al. 2000). Defective huntingtin generated by the HD mutation was suggested to inhibit BDNF transcription (Zuccato et al. 2001). Furthermore, BDNF knockout mice exhibit an earlier age of onset and more severe symptoms (Canals et al. 2004). In the present study, we observed low striatal levels of BDNF after 3-NP intoxication, while rhEPO or IFN-β-1b administration was able to restore BDNF level. In line, a previous study showed that treatment with rhEPO enhances the expression of BDNF in different brain regions of rat (Wang et al. 2016). Similarly, IFN-β-1b therapy was reported to increase BDNF production in multiple sclerosis patients (Mehrpour et al. 2015).

In conclusion, the present study demonstrates the neuroprotective potential of rhEPO or IFN-β-1b on 3-NP-induced behavioral, biochemical, and histopathological alterations in the striatum of rats. These findings might be attributed to improvement of mitochondrial function, anti-oxidant, anti-inflammatory and anti-apoptotic effects. These neuroprotective effects of rhEPO or IFN-β-1b were attributed, at least in part, to the activation of JAK2/STAT3 or JAK1/STAT3, respectively. We also found that early treatment with rhEPO did not confer any benefits compared with late rhEPO treatment, while early IFN-β-1b showed a marked significant benefit especially in reducing neuroinflammation compared with late IFN-β-1b. Thus, either rhEPO or IFN-β-1b may have an immense therapeutic potential in the treatment of clinical HD, but further studies are warranted to confirm these findings.

## Data Availability

Enquiries about data availability should be directed to the authors.
